# Effects of DNA Methylation on Gene Expression and Phenotypic Traits in Cattle: A Review

**DOI:** 10.3390/ijms241511882

**Published:** 2023-07-25

**Authors:** Junxing Zhang, Hui Sheng, Chunli Hu, Fen Li, Bei Cai, Yanfen Ma, Yachun Wang, Yun Ma

**Affiliations:** 1 Key Laboratory of Ruminant Molecular Cell Breeding of Ningxia Hui Autonomous Region, College of Animal Science and Technology, Ningxia University, Yinchuan 750021, China; zhangjunxing@stu.nux.edu.cn (J.Z.); 12021140112@stu.nux.edu.cn (H.S.); 12020131124@stu.nux.edu.cn (C.H.); lifen@nux.edu.cn (F.L.); caibei@nux.edu.cn (B.C.); mayanfen@nux.edu.cn (Y.M.); 2 College of Animal Science and Technology, China Agricultural University, Beijing 100193, China

**Keywords:** cattle, DNA methylation, epigenetic modification, spermatogenesis, embryonic development

## Abstract

Gene expression in cells is determined by the epigenetic state of chromatin. Therefore, the study of epigenetic changes is very important to understand the regulatory mechanism of genes at the molecular, cellular, tissue and organ levels. DNA methylation is one of the most studied epigenetic modifications, which plays an important role in maintaining genome stability and ensuring normal growth and development. Studies have shown that methylation levels in bovine primordial germ cells, the rearrangement of methylation during embryonic development and abnormal methylation during placental development are all closely related to their reproductive processes. In addition, the application of bovine male sterility and assisted reproductive technology is also related to DNA methylation. This review introduces the principle, development of detection methods and application conditions of DNA methylation, with emphasis on the relationship between DNA methylation dynamics and bovine spermatogenesis, embryonic development, disease resistance and muscle and fat development, in order to provide theoretical basis for the application of DNA methylation in cattle breeding in the future.

## 1. Introduction

**Figure 1:**
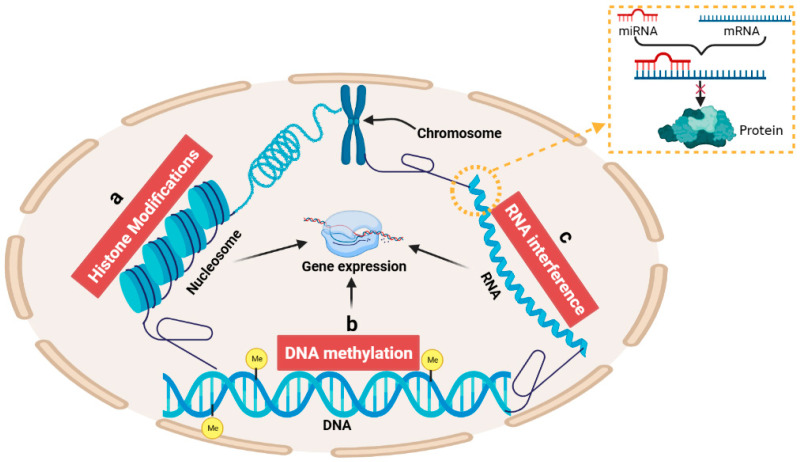
Schematic diagram of epigenetic mechanism. (**a**) histone modification; (**b**) DNA methylation and (**c**) non-coding RNA pathway (mainly microRNA) regulate the transcription process without changing the DNA sequence by regulating the expression of certain genes.

**Figure 2:**
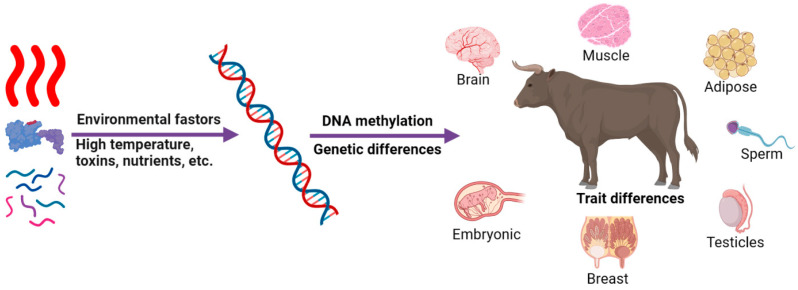
DNA methylation studies in cattle.

**Figure 3:**
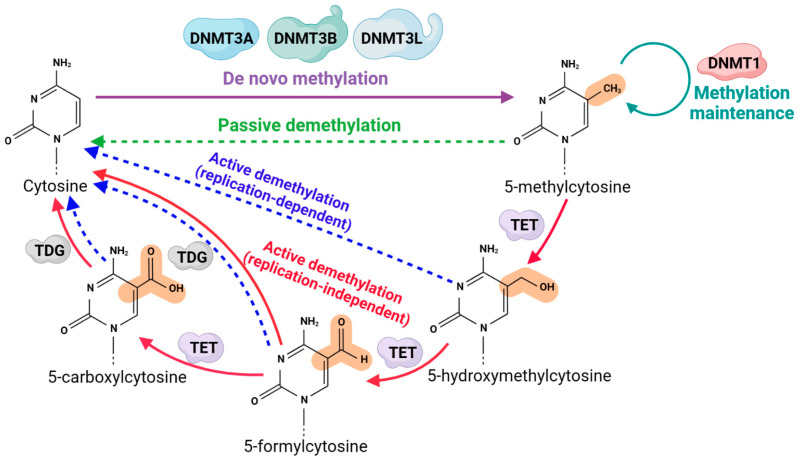
DNA methylation and demethylation mechanisms. DNMT3A and DNMT3B initiate the DNA methylation pattern, DNMT3L can interact with DNMT3A and DNMT3B and stimulate the activity of these two enzymes and DNMT1 participates in the maintenance of DNA methylation pattern. On the other hand, methylated cytosine is converted to unmodified cytosine by active/passive demethylation catalyzed by TET enzyme.

**Figure 4:**
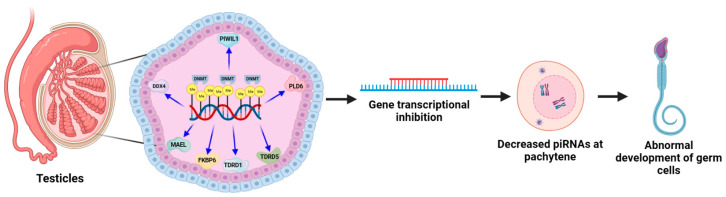
DNA methylation regulates gene expression during spermatogenesis and the production of piRNAs during the thick-line phase through the PIWI/piRNA pathway, leading to failure of germ cell development and affecting bull sterility.

**Figure 5:**
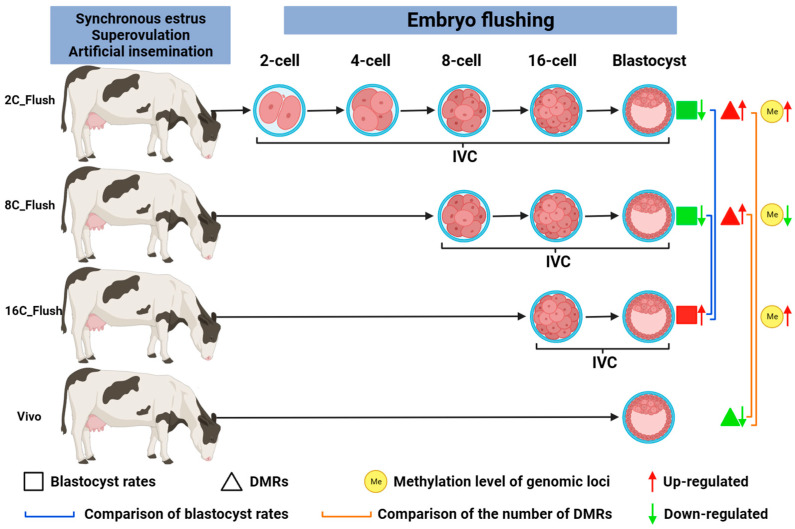
DNA methylation patterns in bovine embryos before, during and after major EGA under in vitro culture conditions. Compared with blastocysts cultured in vivo, the number of DMRs in 2-cell and 8-cell flushed blastocysts increased. Compared with 16-cell flushing group, the blastocyst rate of 2-cell and 8-cell flushing groups was lower. In addition, the number of hypermethylated genomic loci was more than that of hypomethylated loci in blastocysts of 2-cell group and 16-cell group, but the opposite was true in 8-cell group. EGA, Embryonic genome activation. DMRs, Differentially methylated genomic regions.

**Figure 6:**
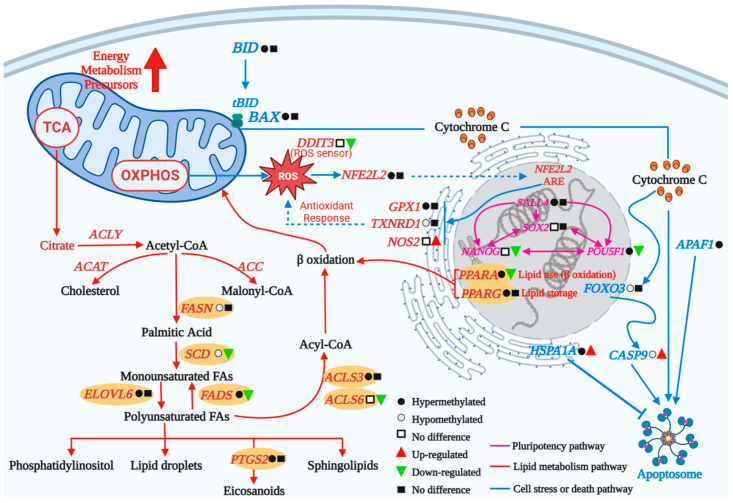
In embryos with different developmental dynamics, the relationship between DNA methylation level and transcriptional status of genes of interest in the biological process containing differential methylation genes was compared to show the importance of these genes to embryonic development and survival. The results showed that there were differences in global DNA methylation profile between fast-dividing embryos and slow-dividing embryos. These differences are mainly related to different metabolic activities, cell structure, survival and death, which may lead to successful or failed pregnancy.

**Figure 7:**
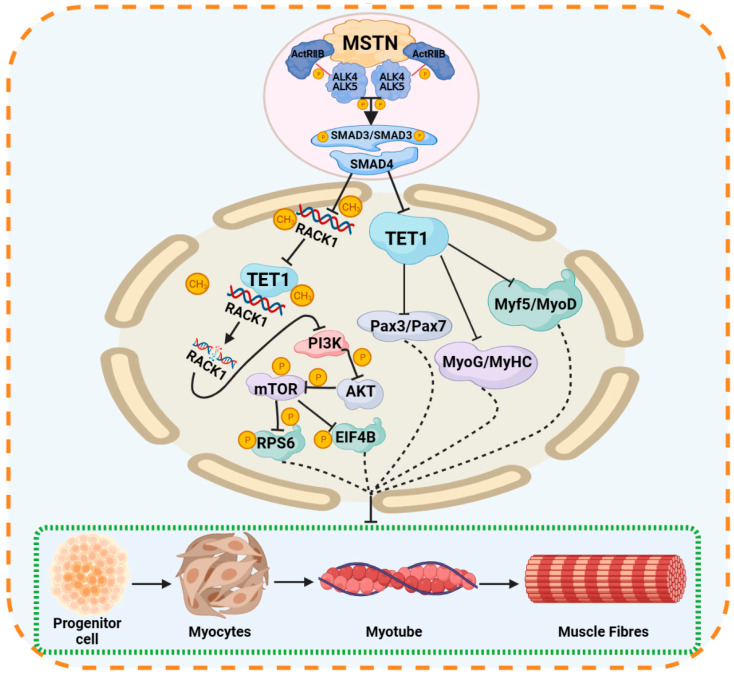
*MSTN* gene regulates skeletal muscle growth and development through DNA methylation pathway. *MSTN* gene inhibits myogenic differentiation of bovine skeletal muscle by increasing the DNA methylation level of RACK1. In addition, *MSTN* mediates the decrease of TET1 transcriptional activity through SMAD3/SMAD3, and then inhibits the demethylation pathway, resulting in a decrease in the transcription level of genes related to myogenic differentiation, and finally negatively regulates the growth and development of bovine skeletal muscle. ‘→’ stands for direct action, ‘- -‘ for indirect action and ‘-|’ for inhibition.

**Figure 8:**
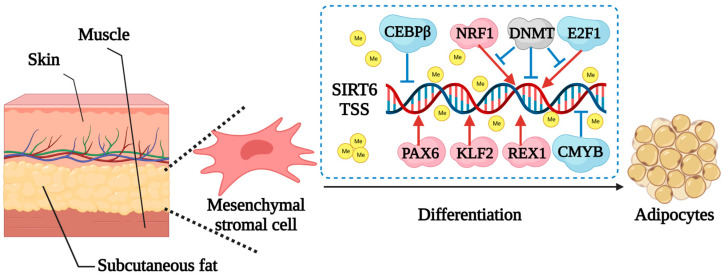
Molecular mechanisms of methylation and transcriptional regulation of *SIRT6* expression in bovine adipocytes. The expression level of SIRT6 was higher in bovine adipose tissue. *PAX6*, *KLF2* and *NRF1* were found to be transcriptional activators of *SIRT6* promoter activity, and *CEBPβ*, *CMYB* and *E2F1* were transcriptional repressors of *SIRT6* promoter activity. In addition, the activity of bovine *SIRT6* promoter is regulated by methylation and the synergistic regulation of *NRF1* and *E2F1* during the differentiation of bovine adipocytes. TSS, transcriptional start site.

**Figure 9:**
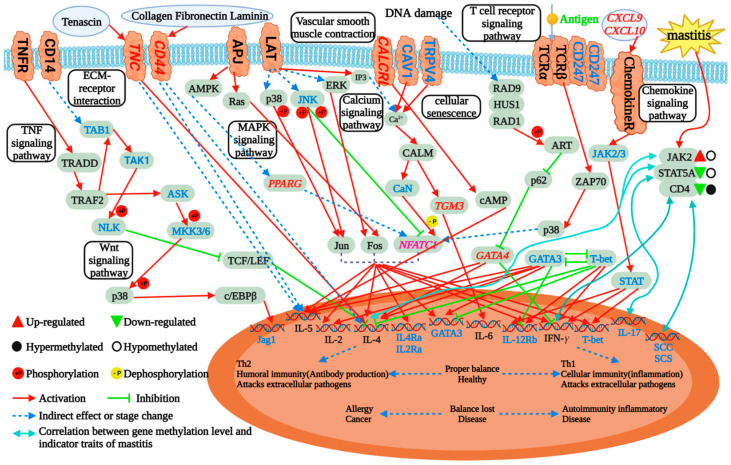
Network of immune-related pathways for mastitis and paratuberculosis. Dairy cow mastitis causes aberration of DNA methylation at CpG site in the 1kb promoter region of *JAK2*, *STAT5A* and *CD4* genes, and the DNA methylation level of these three genes is significantly correlated with the expression level of inflammatory factors. In studies exploring the potential regulatory mechanisms of paratuberculosis in dairy cows, differentially methylated *NFATC1*, *TGM3* and *GATA4* were found to directly influence the expression of immune factors, while *PPARG, CXCL9, CXCL10,* and membrane proteins encoded by *TNC* and *CD44* could indirectly regulate the expression of immune factors through related signal pathway.

Epigenetics is a class of regulatory mechanisms that alter the phenotype of an organism without producing changes in DNA base sequences and that can be passed on to offspring [1]. The investigation of epigenetic alterations holds great significance in comprehending the regulatory mechanism of genes across molecular, cellular, tissue and organ levels. The main mechanisms of epigenetic modification include chromatin remodeling, noncoding RNA regulation, DNA methylation modification and histone modification (acetylation, phosphorylation, ubiquitination, glycosylation) (Figure 1) [2,3,4,5,6,7,8,9,10,11,12]. Among these, DNA methylation is an important epigenetic marker [13]. The level of DNA methylation is affected by RNA interference, abnormal DNA methyltransferase, histone methylation, virus infection, early environmental stimulation, temperature and nutritional supply (Figure 2) [14]. Meanwhile, DNA methylation is time-specific and tissue-specific in the life process of animals and plants, and the degree of DNA methylation varies greatly in different developmental stages. An increasing number of studies have shown that the epigenetics caused by DNA methylation have an important impact on animal reproductive performance [15,16,17,18,19,20,21,22,23,24], growth and development [25,26,27,28,29,30,31,32,33,34,35,36] and disease resistance [15,16,37,38,39], so the methylation of related sites can be used as a molecular marker in future animal breeding (Figure 2, Figure 3, Figure 4, Figure 5, Figure 6, Figure 7, Figure 8 and Figure 9). At present, research on DNA methylation has been extended to the use of hybrid advantage in livestock, molecular genetic breeding and clone optimization and has begun to explore the specific functions of DNA methylation in livestock genetic breeding. This paper provides a review of the role of DNA methylation in the reproduction, development and disease of cattle, providing a theoretical foundation for the progress of cattle breeding and improvement endeavors in a molecular biology context.

## 2. Regulatory Mechanism of DNA Methylation

### 2.1. Catalytic Functions of DNA Methyltransferases

DNA methylation refers to the transfer of methyl to the cytosine of the DNA sequence by the catalysis of DNA methyltransferases (DNMT1, DNMT3A, DNMT3B) to produce 5mC, a modification found mainly in CpG dinucleotides and, to a lesser extent, in non-CpG environments (Figure 3) [40]. DNA methylation is altered at adjacent CpG sites, marked as differentially methylated regions (DMRs) [41]. According to DNMTs’ different functions, they can be divided into two categories: one is brand-new methyltransferases (DNMT3A and DNMT3B), which have no methylation template and can directly catalyze the methylation modification of unmethylated DNA chains; the other is maintenance methyltransferases (DNMT1), which use semimethylated DNA chains as templates to promote methylation of new DNA chains [42]. DNMT3L does not catalyze itself, but it can combine with DNMT3A and DNMT3B to enhance its activity (Figure 3) [43]. The main way of methylation modification in mammals is 5mC. In addition, a small amount of adenosine methylation (N6-mA) also exists, but the mechanism is unclear [40].

### 2.2. Distribution of DNA Methylation in the Genome

DNA methylation mainly occurs at promoters, transposons, enhancers, silencers and gene ontology sites. Methylation occurs at approximately 70% of CpGs in the genome, whereas unmethylated CpGs cluster to form CpG islands located at core sequences of gene promoters and transcription start sites [44]. CpG islands are mostly found in the promoter regions of developmental and housekeeping genes. The occurrence of DNA methylation in this region can make methyl CpG binding region proteins hinder the binding of transcription factors and RNA polymerase to the template strand. This inhibition of the transcription process alters gene expression levels, ultimately leading to changes in the corresponding biological functions of the organism [45]. The DNA methylation of transposons highly inhibits their transposable activity, thus maintaining the stability of the genome. The researchers identified and characterized the pilRNAs present in bovine gametes, gonads and zygotes. These pilRNAs exhibited several canonical characteristics of piRNAs, including a 1U bias, the presence of a ‘ping-pong’ signature, genomic clustering and the targeting of transposable elements. Among the transposons targeted by pilRNAs in oocytes and zygotes, the LINE RTE and ERV1 classes were prominent. Additionally, pools of pilRNAs potentially derived from or targeting specific mRNA sequences were identified [46]. The DNA methylation status of enhancers is negatively regulated with gene activity, while the DNA methylation status of silencers is positively correlated with gene activity. Thousands of genomic regions associated with H3K27ac modification, known as active enhancers, were identified in bovine satellite cells before or during differentiation. These enhancer regions contain binding sites for various transcription factors, including MYOG and MYOD1 [47]. DNMT1 silencing significantly decreased the methylation levels of miR-29b promoter, up-regulated miR-29b expression and inhibited BVDV NADL replication, which supports the important roles of DNA methylation in regulating miRNA expression [48]. The relationship between the DNA methylation of gene ontology and gene expression may vary with different species or cell types. A global map of regulatory elements (15 chromatin states) was established in cattle, with their coordinated activities defined [49]. This was achieved through the genome-wide profiling of six histone modifications, RNA polymerase II, CTCF-binding sites, DNA accessibility, DNA methylation and transcriptome analysis in rumen epithelial primary cells (REPC), rumen tissues and Madin-Darby bovine kidney epithelial cells (MDBK). The study demonstrated that each chromatin state displayed specific enrichment patterns in sequence ontology, transcription, methylation, trait-associated variants, gene expression-associated variants, selection signatures and evolutionarily conserved elements [49].

### 2.3. Regulatory Mechanism of DNA Methylation on Gene Expression

DNA methylation and demethylation are complex regulatory processes that could affect the selective expression of genes (Figure 3) and ultimately determine growth and organ differentiation. At present, the main regulatory mechanisms of DNA methylation on gene expression have been concluded in the following: (1) Affecting the conformation of DNA molecules: DNA methylation can not only affect the interaction between gene transcription factors and the upstream regulatory region of the gene but also indirectly mediate transcriptional inhibition by changing the structure of chromatin. (2) Imposing a steric hindrance effect: The methyl group of 5-methylcytosine is located in the DNA sulcus, which can prevent the binding of transcription factors to DNA, thus interfering with the normal function of transcription factors. (3) Facilitating protein specificity: When the CpG site is methylated, regulation of gene expression is achieved through the recruitment and binding of specific proteins. (4) Affecting the transcriptional level: RNA-mediated DNA methylation leads to gene silencing at the transcriptional level, especially methylation in the promoter region, which negatively regulates gene expression. Guo et al. discovered the crystal structure of DNMT3A (ADD and catalytic domain) and DNMT3L (catalytic domain-like) in a self-inhibited conformation when not bound to the unmodified H3 tail [50]. Upon binding of DNMT3A to unmethylated H3, the interaction between the ADD and catalytic domains is disrupted, allowing for DNA binding and subsequent methylation. Some studies have found that DNMT3B is specifically recruited to active transcription genes through its PWWP domain, which interacts with H3K36me3 [51]; DNMT3A is specifically recruited to intergenic regions by H3K36me2 [52]. DNA sequence and shape readout are key factors in achieving transcription factor binding specificity. CpG methylation significantly alters the local DNA shape, which can potentially impact the accessibility of transcription factors to target sequences [53].

## 3. DNA Methylation Detection Methods

### 3.1. Common Methods for DNA Methylation Detection

The DNA methylation detection methods can be categorized into the following three areas: (1) Studies at the whole genome level through various techniques, including DNA methylation immunoprecipitation (MeDIP-seq) [54], whole genome bisulfite sequencing (WGBS) [55], next generation sequencing (NGS) [56], and nanopore sequencing [57,58]. (2) Studies based on investigations of methylation-specific sites, in which bisulfite sequencing PCR (BSP) [59], methylation-specific PCR (MS-PCR) [60], combined bisulfite restriction analysis (COBRA) [61,62], methylation-sensitive high-resolution amplification (MS-HRM) [63], methylation-sensitive single nucleotide primer amplification (MS-SnuPE) [64,65], reduced representation bisulfite sequencing (RRBS) and oxidative-reduced representation bisulfite sequencing (oxRRBS) [66,67] are used. (3) Studies based on investigations of new methylation sites including various techniques, such as MBD column chromatography [68] and DNA microarray [69].

### 3.2. Advantages and Limitations of Common Detection Methods

Each detection method has its own advantages and limitations (Table 1). For example, MeDIP-seq uses specific antibodies or proteins to capture, purify, amplify and sequence methylated DNA fragments and estimate the methylation level of a specific region according to the results of reference genome mapping, but this method cannot accurately identify methylation sites [70,71]. NGS makes it possible to avoid the distortions associated with the use of specific probes, allelic differences and amplification in microarray technology, enables the assessment of DNA methylation at single base resolution and has the potential to provide rapid access to complex data on DNA methylation in mapped genomic species [56,72]. Nanopore sequencing is the fourth generation of DNA sequencing technology and can identify DNA methylation patterns without PCR amplification or the chemical labeling of samples and can record the ion current deviation caused by specific base modifications through pores [73,74]. The main disadvantage of this technique is that the error rate is relatively high. So, the bioinformatics tools and algorithms used are constantly improved and optimized [75,76,77,78]. MS-PCR is a methylation analysis method applicable only to CpG islands; it cannot be used to detect sites with low numbers of CpGs, and the design of primers is relatively difficult [79]. RRBS reduces the number of reads required by focusing only on genomic segments containing CpG islands (CGIs) but ignores the detection of methylation levels in non-CGI regions [80,81,82]. COBRA is a technique that combines restriction endonuclease with bisulfite transformation [62,83]. This method has software support (methyl typing) but is limited by the restriction site, and its program is time-consuming. When the sample is not digested completely, it will produce false-positive results. MS-HRM is a method based on the analysis of different temperatures between C-G (3 H bonds) and A-T (2 H bonds) pairs [84,85]. When methylation occurs in only a small proportion of the sample sites analyzed, the method makes it possible to discriminate between fully or partially methylated sites and unmethylated sites with high efficiency. The disadvantage is that it is not possible to analyze only one CpG. The advantages of MS-SnuPE include single cytosine analysis that does not require restriction enzymes or sequencing, semiquantitative analysis and the fact that multiple CpGs can be analyzed per reaction using multiple strategies [86,87,88]. The disadvantage is that each site requires two parallel reactions and includes radio-labeled compounds. WGBS is the gold standard of DNA methylation detection and can provide a complete map of genome-wide 5mC. However, its use is limited by high cost, tedious analysis and a small number of project samples [89].

### 3.3. Some New Techniques for DNA Methylation Detection

There have been a large number of techniques available for DNA methylation analysis, but these techniques have some limitations. Therefore, researchers are working to develop new tools. In recent years, scientists have focused on the development of alternative detection methods in the form of biosensors, which they divided into optical biosensors and electrochemical biosensors according to signal transduction. The optical biosensor detects the light generated when the target analyte is captured by the biometric layer, while the electrochemical biosensor detects the changes in electrical parameters before and after biometric recognition of the target analyte [90,91]. Among these methods, CRISPR-Cas-assisted sensor systems have shown great promise and attracted widespread interest in site-specific DNA methylation detection based on their high specificity of binding and signal amplification capabilities [92]. However, although these biosensors have the advantages of simplicity, low cost, high sensitivity and high detection efficiency, they have limitations in the application of clinical samples [93]. In addition, emerging single-cell DNA methylome sequencing technologies have begun to characterize dynamic methylation events in highly heterogeneous cell populations [94]. This approach has increased experimental throughput and genomic coverage but suffers from high implementation costs and low yields. Extensive crosstalk between DNA methylation and histone modification can form a dynamic methylation landscape and promote the gene regulation of different molecular processes [94]. To better understand the crosstalk between the two epigenetic regulation mechanisms, researchers have developed multiple groups of methods based on single-cell DNA methyl group analysis to study cellular heterogeneity at different time scales to reveal new relationships between genome/epigenetic regulation and its functional output [95,96,97,98,99,100,101]. However, these sequencing methods generally depend on the bisulfite conversion reaction and cannot distinguish between 5mC and 5hmC [94]. Therefore, it is still the focus of future research to improve the existing technologies or combine different methods to develop new techniques and obtain specific, sensitive, easy-to-operate and high-throughput assays.

**Table 1:** DNA methylation detection methods.

Level	Method	Technical Complexity	Cost	Specificity	Large Sample Sizes	References
the whole genome level	MeDIP-seq	Moderate	High	Moderate	Relatively friendly	[54,70,71]
	WGBS	High	High	Very high	Not be as friendly	[55,89]
	NGS	Moderate	High	Depends on the design	Generally friendly	[56,72]
	nanopore sequencing	Moderate	Moderate	High	Relatively friendly	[57,58,73,74,75,76,77,78]
methylation-specific sites	BSP, MS-PCR	Low	Low	High	Relatively friendly	[59,60,79]
	COBRA	Moderate	Moderate	High	Relatively friendly	[61,62,83]
	MS-HRM	Moderate	Moderate	High	Relatively friendly	[63,84,85]
	MS-SnuPE	Moderate	Moderate	High	Relatively friendly	[64,65,86,87,88]
	RRBS, oxRRBS	Moderate	High	High	Not be as friendly	[66,67,80,81,82]
	CRISPR-Cas-assisted sensor systems	High	High	High	Not be as friendly	[92,93]
new methylation sites	MBD column chromatography	Moderate	Moderate	High	Relatively friendly	[68]
	DNA microarray	Moderate	Moderate	Depends on the design	Relatively friendly	[69]

## 4. The Biological Role of DNA Methylation

The difference in phenotype is affected by heredity and environment [102]. Epigenetic modification affects the cell phenotype by affecting gene expression, which is inherited by progeny cells during mitosis, and then progeny cells have the same epigenetic markers as parental cells [103]. In addition, DNA methylation status can regulate gene expression profiles, resulting in different phenotypes, which might further influence productivity and disease risk [104,105].

### 4.1. Studies on DNA Methylation Associated with Reproduction in Cattle

The *KPNA7* gene, a maternal factor specific to oocytes, controls the transport of nuclear proteins and functions during early embryonic development [106]. Three oocyte-specific differentially methylated CpG sites were found by comparing DNA methylation profiles proximal to the *KPNA7* gene promoter in oocytes and six different somatic cells. The results indicate that the restricted expression of the bovine *KPNA7* gene in oocytes is regulated by DNA methylation of its proximal promoter and that demethylation of three CpG sites was closely associated with the tissue specificity of the *KPNA7* gene [106]. Rekawiecki et al. assessed the percentage of methylation in the promoters of progesterone receptor isozymes A (PGRA) and B (PGRB) and determined that the hypermethylation level of the promoter region of the PGRA subtype might be a mechanism for regulating the inhibition of PGRB activity by PGRA, which may affect the regulation of progesterone in the luteum and endometrium [107]. Takeda used frozen semen samples from Japanese black bulls to record the sire conception rate (SCR), and a human methylation gene chip was used to analyze the methylation level of each CpG site [108]. The results showed significant differences in the methylation of 143 CpG loci related to SCR. At the same time, the differential methylation region (DMRs) of the target CpG site was identified, and fertility-related methylation changes were detected in 10 DMRs. Finally, through multiple regression analysis of methylation level and SCR, three DMRs that can effectively predict the fertilization rate of bulls were screened. It was concluded that these differences in sperm methylation levels related to fertility may be new epigenetic biomarkers for predicting bull fertility [108]. In Table 2, we also listed other studies related to cattle reproduction.

The heterosis of interspecific hybridization between cattle and yak could increase body size and improve milk production performance, but there is a decline in fertility performance, resulting in their dominance not being maintained in the offspring [112]. Immunohistochemical and image analysis techniques were used to detect the expression of 5mC and acetylated histone H3 lyase (AcK9) in spermatogonia and testicular cells of hybrid cattle and yak [112]. Compared with yaks of the same age, all cell types of hybrid cattle showed higher levels of 5mc expression and different degrees of Ack9 expression, indicating that DNA methylation and the inappropriate expression of AcK9 may be the main factors of male sterility in hybrid cattle. Li et al. found that the expression of *PRDM9* mRNA in the testes of hybrid yaks and immature calves was significantly lower than that of adult yaks, while the low expression of *PRDM9* could inhibit the activity of histone methyltransferase and affect the process of cell meiosis, resulting in sterility in hybrid bulls [113]. Epigenetic modifications of DNA methylation and P-element-induced weak testis-interacting RNAs (piRNAs) are important regulators in spermatogenesis. It was found that yak male infertility is associated with promoter hypermethylation and the transcriptional silencing of the piwi/piRNA pathway-associated genes *TDRD1*, *PLD6*, *PIWIL1*, *DDX4*, *MAEL*, *FKBP6*, and *TDRD5*, and the transcriptional silencing of these genes drives the reduced production of piRNAs in the pachytene stage of cattle germ cells, ultimately resulting in the failure of germ cell development (Figure 4) [114]. The bull used for artificial insemination is a good model to study the mechanism of male infertility and spermatogenesis defects. The DNA methylation profiles of semen samples from 100 bulls of the same age were detected according to the infertility rate after insemination [115]. The results identified 490 differentially methylated cytosines associated with fertility, and most of these differentially methylated cytosines were in a hypermethylated state in infertile bulls. In addition, 46 genes targeted by differentially methylated cytosines were found to be related to sperm function, maturation and embryonic development, indicating that sperm DNA methylation status can be used as a biomarker for the identification of male fertility. In summary, constantly exploring the relationship between the DNA methylation level of imprinted genes and male sterility will become a new way to address the sterility of bulls.

### 4.2. Studies on the Relationship between DNA Methylation and Bovine Embryonic Development

There are two types of reprogramming of DNA methylation during embryonic development, which occur at the stage of gametogenesis and the early stage of embryonic development [116]. The first reprogramming is necessary for the removal and re-establishment of parental imprinted genes, and the second is necessary for fertilized oocytes to acquire totipotency and produce new individuals. Meanwhile, the same sequence could possess different DNA methylation patterns in different tissues, regulating the specific expression of genes [117]. In addition, epigenetic modifiers such as methionine, choline, folic acid and vitamin B12 have been proven to participate in methylation responses during early embryonic development [118].

Abnormal DNA methylation patterns of genes required for development are common in-vitro-produced embryos. Under in vitro culture conditions, the DNA methylation patterns of bovine embryos before, during and after main embryonic genome activation (EGA) were studied. It was found that the probability of 2-cell and 8-cell embryos growing to the blastocyst stage in vitro was lower than that of 16-cell embryos (Figure 5) [119]. In addition, compared with the control group cultured completely in vivo, the number of DMRs in 2-cell and 8-cell blastocysts cultured in vitro also increased, and the number of genomic loci with low methylation was more than that with hypermethylation in 8-cell blastocysts (Figure 5) [119]. Taken together, these results suggest that in vivo embryos cultured in vitro before and during EGA are more susceptible to alterations in DNA methylation marks and that transferring in vivo embryos during EGA to in vitro culture increases hypomethylation motifs in blastocysts (Figure 5) [119]. Ispada et al. analyzed the methylation of different genomic sequences in bovine blastocysts from fast (four or more cells) and slow (two cells) embryos, which was used to compare the overall DNA methylation profiles of embryos with different developmental dynamics and thus to identify the regions and pathways most affected by this phenotype (Figure 6) [120]. The results showed that there were more (7976) hypermethylated regions in rapid embryos, which were mainly distributed in the genome and included regions such as promoters, exons, introns and repeating elements. In contrast, 3608 hypermethylated regions were identified in slow embryos, which were more frequently found on CpG islands. In addition, DMRs were mainly concentrated in pathways related to cell survival/differentiation and energy/lipid metabolism, indicating that the dynamics of the first cleavage will affect the level of DNA methylation and the expression profile of genes related to metabolism and differentiation pathways and may affect the survival rate of embryos (Figure 6) [120]. The semen of high-fecundity bulls and low-fecundity bulls was collected, and the methylation patterns of embryos produced by semen fertilization were compared. The results showed that there was no difference in the morphology and developmental ability of embryos produced by the semen of high and low-fecundity bulls, but 76 differential methylation regions were revealed in the evaluation of the epigenetic characteristics of sperm [121]. Comparing the level of DNA methylation in the first cell cycle of bovine in vitro fertilization (IVF) and bovine somatic nuclear transfer (SCNT) embryos [122], bovine SCNT embryos underwent abnormal epigenetic reprogramming in the first cell cycle and had more significant demethylation and higher remethylation than bovine IVF embryos, indicating that different methods of nuclear transfer could affect the difference in DNA methylation of embryos. ZFP57 is a key protein needed for imprinting maintenance after fertilization, and its expression can maintain normal methylation in the imprinting control region. Yu et al. found that the methylation level of the imprinting control region of key imprinted genes in SCNT-cloned embryos was lower than that of in vitro fertilization embryos, indicating the loss of imprinted gene methylation [123]. Increasing the expression of ZFP57 in donor cells promoted the development of SCNT blastocysts, increased the number of trophoblast cells and total cells, decreased the rate of apoptosis and achieved a degree of methylation similar to that of in vitro fertilized embryos, which provided an effective means to enhance the nuclear reprogramming and developmental potential of SCNT embryos. Therefore, many studies on embryonic DNA methylation can not only elucidate the epigenetic modification mechanism in embryonic development but also provide help for early embryonic abnormal development screening and timely correction of abnormal methylation.

### 4.3. DNA Methylation Associated with Bovine Muscle Development in Cattle

Wang et al. detected the level and expression of DNA methylation in bovine muscle tissue and found that the DNA methylation pattern had a significant effect on the level of mRNA in muscle tissue [124]. Genome-wide methylation profiles and transcriptional profiles in the longest dorsal muscle tissue of yaks at different developmental stages (90 days of age, 6 months of age and 3 years of age) revealed that methylation levels in the promoter regions of the muscle development-related genes *CACNA1S*, *IGF2*, *MUSTN1* and *TMEM8C* were negatively correlated with gene expression and affected muscle development and meat quality [26]. Genome-wide methylation profiles of fetal and adult longest dorsal muscles of Chinese Qinchuan cattle were constructed, and 77 genes were found to be affected by methylation levels. The expression levels of the high methylation genes *LAMB1*, *HNRNPM*, *ACLY*, *CLCN2*, *CRABP2*, *ADAM12*, *MBOAT2* and *PSD* were reduced in adult muscle tissue, and the expression levels of the low methylation genes *MYL2*, *PDLIM1*, *DUSP1*, *DTNBP1*, *CS* and *EEF1A2* were increased in adult muscle tissue [125]. The transcriptional level of the *FoxO1* gene is regulated by methylation in the promoter region. For instance, previous studies have shown that the methylation level of the *FoxO1* gene in the promoter region was significantly higher in calves than in adult cattle, and the gene expression level was significantly lower [29]. In addition, silencing the expression of *FoxO1* can promote the proliferation and differentiation of bovine myoblasts, indicating its negative regulation in the proliferation and differentiation of bovine myogenic cells [29]. Myostatin (*MSTN*) is a cytokine that negatively regulates the growth and development of skeletal muscle [126]. The expression of *MSTN* can cause an increase in DNA methylation and inhibit the binding of the demethylase TET1 to the *RACK1* promoter region, resulting in hypermethylation and decreased transcription levels in the *RACK1* promoter region (Figure 7) [127]. The reduced *RACK1* further inhibits the myogenic differentiation of bovine skeletal muscle satellite cells by inhibiting the PI3K/AKT/mTOR pathway (Figure 7) [127]. In addition, *MSTN* increases the methylation level of myogenic-specific genes by down-regulating the expression of TET1, which is directly controlled by SMAD1/SMAD2, and ultimately inhibits skeletal muscle growth and development (Figure 7) [128]. In summary, DNA methylation plays an important role in the regulation of muscle growth and development.

### 4.4. Studies on DNA Methylation Associated with Fat Deposition in Cattle

Retinoic acid can regulate cell differentiation and proliferation by regulating the transcription of multiple target genes. Retinoic acid binding protein 2 (CRABP2) was a candidate gene affecting beef quality, yield and fat deposition [129]. The expression and methylation patterns of the *CRABP2* promoter differential methylation region (DMR) in Jiaxian red bull, Jiaxian red bull × Angus and Jiaxian red bull × Simmental were studied [129]. It was found that there were different methylation and expression patterns of *CRABP2* in different breeds and different tissues, and the methylation state of *CRABP2* DMR could regulate the expression of other genes, which could be an important parameter for studying beef quality traits [129]. *SIRT6* is a regulator of lipid metabolism, insulin secretion and glucose metabolism. The expression and the methylation level of the core promoter region showed that it is highly expressed in bovine subcutaneous adipose tissue, and the activity of the *SIRT6* promoter in adipocytes was regulated by methylation (Figure 8) [130]. The significant differences in meat quality traits between China Red Steppe cattle and Japanese black cattle provide a model for investigating the mechanisms by which DNA methylation regulates meat quality traits. Fang et al. conducted a genome-wide DNA methylation analysis on both breeds and identified 23,150 DMRs out of 8596 genes, which were mainly enriched in the lipid transport and lipid translocation pathways [30]. In addition, correlation analysis identified 331 DMRs that were negatively correlated with the expression of differentially expressed genes (DEGs), 21 of which were located in the promoter region, which will provide a basis for marker-assisted selection in studies of meat quality traits [30]. Wang et al. analyzed DNA methylation patterns in the breast tissues of 17 Canadian Holstein cows with different milk fat contents and found 706 differentially methylated CpG loci (DMCs) [131]. Among them, 83 DMCs and 87 quantitative trait loci (QTLs) were colocalized in milk composition and yield traits, indicating that these methylation changes potentially participate in the regulation of milk fat content [131].

### 4.5. Studies on DNA Methylation Associated with Resistance Performance in Cattle

The DNA methylation level has a correlation with the environment and disease resistance in cattle (Figure 2 and Figure 9) [15,16,17,37,38,39]. In the study of genomic DNA methylation in blood samples of heat-tolerant bulls and Angus bulls, it was found that heat stress can cause variation in methylation patterns of specific loci, which provides a theoretical basis for further study of the role of DNA methylation in heat tolerance in cattle [132]. In a study comparing DNA methylation patterns in Indian cattle (*Bos indicus*) and crossbred cattle, there were differences in the expression patterns of DNA methyltransferases (DNMT1, DNMT3A) and demethylases (TET1, TET2 and TET3). Low methylation levels promoted the expression of the stress genes *HSP70* and *STP1*, suggesting a role for DNA methylation in the regulation of the heat stress response pathway [133]. DNA methylation is also involved in the occurrence of dairy cow mastitis. In the breast tissue of dairy cows with clinical mastitis, the level of DNA methylation in exon 2 of the bovine *IL6R* gene is up-regulated, and it is speculated that its methylation levels may be a potential biomarker for monitoring dairy cow mastitis [134]. Comparing the total DNA methylation level of neutrophils in blood between healthy cows and cows with mastitis, it was found that the methylation level of neutrophils in cows with mastitis decreased [135]. Meanwhile, it was found that the methylation status of the promoters of the *CITED2* and *SLC40A1* genes, the key regulators of inflammation, would affect their differential expression, and the methylation of exon 5 of the *LGR4* gene could regulate their own alternative splicing [135]. In addition, abnormal DNA methylation at the CpG site in the 1 kb promoter region of *JAK2*, *STAT5A* and *CD4* genes caused by mastitis can be used as a potential epigenetic marker to estimate mastitis susceptibility in dairy cows (Figure 9) [16]. The expression of hypoxia-inducible factor HIFs in several tissues of yak and other low-altitude cattle was analyzed by Xiong, and the methylation status of the promoter region was detected [136]. It was found that the methylation level of *HIF-1α* and the *HIF-2α* 5′ flanking regulatory region in the kidney tissue of yaks was significantly lower than that of cattle at low altitude, indicating that the hypoxia stress response at high altitude was related to the difference in body methylation level [136]. In summary, DNA methylation-related genes can be used as genetic markers for animal disease-resistance breeding and, combined with modern molecular marker technology, will greatly improve the efficiency of stress-resistant breeding and provide strong support for the selection of stress-resistant varieties.

## 5. Conclusions and Future Perspectives

DNA methylation is a highly conserved epigenetic modification that plays an important role in gene regulation, genome stability and development in mammals. With the progress of DNA sequencing technology, the number of studies exploring the role of DNA methylation in bovine reproduction, growth and development, and resistance is increasing. These studies have analyzed the genome-wide regulation of DNA methylation on gene expression, found some new important genes related to differential methylation regions and ways that may affect important economic traits of cattle and investigated the changes in methylation patterns under the influence of the environment, development and disease. In addition, in the latest study of DNA methylation inheritance, researchers divided DNA methylation inheritance into intergenerational inheritance and transgenerational inheritance and found that environmental factors may induce heritable phenotypes in cattle in a non-genetic way [137,138,139,140,141,142]. Genetic studies of DNA methylation during mitosis in mammalian cells have shown that DNA methylation acts through the establishment of stable genetic epigenetic memory and is stably propagated during cell division, but DNA methylation may also be dynamic in some key regulatory elements, and the kinetics of methylation turnover can influence gene expression [143]. In the evaluation of heritability and age effect of DNA methylation level at specific sites and cumulative DNA methylation load at all sites in cattle, it was found that the DNA methylation map was determined by genetic and environmental factors (such as the age of beef cattle) [144]. Once inherited, DNA methylation patterns undergo changes throughout life under environmental influences and aging-relevant processes [145]. Zhou et al. analyzed the DNA methylomes of 16 major bovine tissues by WGBS, studied the landscapes of DNA methylomes in different tissues, compared their differences in many cases and constructed the largest bovine DNA methylation epigenome data set so far, which provided comprehensive resources for the study of bovine epigenome [146]. These results are of far-reaching significance for understanding the complex biological characteristics of important economic traits and will provide a very powerful theoretical basis for subsequent genetic improvement in cattle breeding. In addition, dynamic modifications in DNA methylation have been found to play an essential regulatory role in studies of bovine embryonic development. If DNA methylation is abnormal, it may cause embryo development obstruction or even malformation or death, which is one of the main reasons for the low survival rate of SCNT and in vitro reproductive embryos. Therefore, an in-depth study of DNA methylation and understanding the abnormal genome-wide dynamic pattern can provide a reference for improving the survival rate of bovine embryos.

With the development of DNA methylation detection technology, we have obtained a large number of base-resolved methylation maps, mastered the knowledge of the key factors in establishing, maintaining and eliminating DNA methylation and revealed a series of basic biological functions. However, current research still faces many challenges, such as the difficulty in obtaining certain tissue and disease samples; the difficulty in establishing stable reference models for DNA methylation due to the dynamic and relatively unstable nature of DNA methylation and the question of how methylation can be practically applied to reproduction and breeding efforts. These are all questions that need to be explored in the future. We believe that with the establishment of DNA genome-wide methylation bioinformatics libraries and the development of DNA methylation detection technology at a later stage, these problems will be gradually resolved. Overall, DNA methylation is an important part of genetic research and may help in the selection and breeding of cattle.

## Figures and Tables

**Table 2:** DNA methylation affects offspring phenotypes by regulating related traits in cattle spermatozoa.

Breed	Period	Sample	Phenotype	Influenced Offspring Phenotype	Reference
Norwegian Red bull	459–517 days	16 semen	56-day non-return rates	chromatin integrity traits, in vitro embryo development	[109]
Holstein bull	Adult	6 semen	age, sire conception rate	sire conception rate	[110]
Holstein bull	1–2 years old	28 semen	sperm quality	daughters’ reproductive traits	[23]
Holstein bull	Adult	2 semen	sperm quality	daughters’ reproductive traits	[111]

## Data Availability

All data are reported in this manuscript.
